# Cytokine Storm Induction Linked to Multi‐Organ Failure in Fatal Jellyfish Stings

**DOI:** 10.1002/advs.202501104

**Published:** 2025-07-13

**Authors:** Yichao Wang, Yi Wang, Fengling Yang, Jingbo Chen, Xiaoyu Geng, Qing Sun, Jinyu Zhang, Chang Liu, Jie Lv, Xiaochuan Hou, Yongfang Wang, Huiyan Lin, Jing Zhang, Lingxin Chen, Bing Yan, Liang Xiao

**Affiliations:** ^1^ Faculty of Naval Medicine Naval Medical University Shanghai 200433 China; ^2^ Department of Clinical Laboratory The First People's Hospital of Xianyang Xianyang Shannxi 712000 China; ^3^ College of Traditional Chinese Medicine Jilin Agricultural University Changchun Jilin 130118 China; ^4^ Key Laboratory of Biological Defense Ministry of Education Second Military Medical University Shanghai 200433 China; ^5^ Shanghai Key Laboratory of Medical Bioprotection Second Military Medical University Shanghai 200433 China; ^6^ Department of Military and Special Medicine No.971 Hospital of the PLA Navy Qingdao Shandong 266071 China; ^7^ Department of Intensive Care Medicine No.971 Hospital of the PLA Navy Qingdao Shandong 266071 China; ^8^ CAS Key Laboratory of Coastal Environmental Processes and Ecological Remediation Research Center for Coastal Environmental Engineering and Technology Yantai Institute of Coastal Zone Research Chinese Academy of Sciences Yantai Shandong 264003 China; ^9^ Institute of Environmental Research at the Greater Bay Area Key Laboratory for Water Quality and Conservation of the Pearl River Delta Ministry of Education Guangzhou University Guangzhou Guangdong 510006 China

**Keywords:** cytokine storm, envenomation, inflammation, jellyfish, NF‐κB p65

## Abstract

Fatal jellyfish stings often cause multi‐organ failure. Until now, these fatal outcomes are attributed to the direct toxic effects of the venom. Here, a mouse model of delayed jellyfish envenomation syndrome (DJES) is established and showed that venom from Nemopilema nomurai jellyfish can trigger a deadly cytokine storm – a severe inflammatory reaction. Mice injected with the venom displayed acute multi‐organ failure and significant upregulation of over 20 pro‐inflammatory cytokines (including IL‐6, TNF‐α, CXCL2, and CCL4) in the heart, liver, and kidneys. Transcriptomic analyses identified NF‐κB p65 subunit activation as central to the cytokine storm induction. Knockdown of p65 in macrophages reduced cytokine production and improved cell viability. Treatment with dexamethasone, an NF‐κB inhibitor, effectively suppressed the cytokine storm, mitigated organ damage, and increased survival rates in mice. The findings present new insights to treat fatal jellyfish stings.

## Graphical abstract

*N. nomurai* is the most important injurious species in the coastal area of China. Model construction, omics analysis, mechanism exploration, and intervention measures of *N. nomurai* envenomation are performed in the current study, and it suggested that cytokine storm played an important role in pathogenesis. These findings present new insights to treat fatal jellyfish stings.

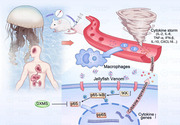

## 1 Introduction

Jellyfish stings affect ≈150 million people yearly and account for 90% of marine organism‐related injuries.^[^ ^1^, ^2^ ^]^ These incidents are prevalent during summer when jellyfish populations surge in the coastal regions of Australia,^[^ ^3^ ^]^ Japan,^[^ ^4^ ^]^ and China.^[^ ^5^ ^]^ Global warming and coastal water eutrophication^[^ ^6^ ^]^ have increased the virulence of the stings and prevalence of dangerous jellyfish species such as *Nemopilema nomurai* (also known as *Stomolophus meleagris*), *Cyanea capillata*, *Chironex fleckeri*, and *Physalia physalis* ^[^ ^3^, ^5^ ^]^ that are responsible for thousands of envenomation cases annually. Symptoms from these stings range from mild local irritation to severe systemic reactions^[^ ^7^ ^]^ that can escalate to edema, myalgia, dyspnea, and potentially fatal profound cardiovascular collapse.^[^ ^3^ ^]^

Depending on the species, jellyfish stings can cause either hyperacute fatalities, acute fatalities, or Delayed Jellyfish Envenomation Syndrome (DJES)^[^ ^8^, ^9^ ^]^ The first two cases are mainly due to the venom's components, such as porin,^[^ ^10^ ^]^ phospholipase,^[^ ^11^ ^]^ and cardiotoxin,^[^ ^12^ ^]^ which can directly damage ion channels in the cardiovascular^[^ ^13^ ^]^ and nervous systems,^[^ ^14^ ^]^ leading to death in an instant or within a few hours.^[^ ^8^ ^]^ In contrast, DJES is characterized by a delayed immune response, with symptoms appearing only days after the sting,^[^ ^9^ ^]^ while DJES can be indirectly triggered by low doses of jellyfish toxins, which are insufficient to cause immediate toxicity but are adequate for initiating an exaggerated immune response that can lead to severe multi‐organ damage.^[^ ^15^ ^]^ However, exactly how minimal venom exposure precipitates DJES^[^ ^16^ ^]^ and the overactivated immune response is poorly understood.^[^ ^17^, ^18^, ^19^ ^]^ The delayed immune reaction also suggests that there may be a critical window for medical intervention to prevent severe outcomes.^[^ ^20^ ^]^

Our results characterize DJES by two major clinical phases: target cell and tissue injury, followed by a severe inflammatory response with overproduced cytokines termed “cytokine storm,” This condition often occurs after systemic infections like sepsis, immunotherapies, and exposure to toxins. The overall levels of cytokines such as IL‐6, CCL2, G‐CSF, IL‐3, IFN‐γ, GM‐CSF, and IL‐2 correlate strongly with the severity of cytokine storms and mortality rates. For example, IFN‐γ and TNF have been extensively studied for their roles in cell death, inducing necroptosis or apoptosis independently, and are linked to mortality in conditions like COVID‐19, as well as numerous pathological conditions, including neurological disorders.^[^ ^21^ ^]^

Here, we show that the venom from *N. nomurai* jellyfish triggers a cytokine storm in a DJES mouse model established in our lab. We replicated the acute multi‐organ failure observed in a patient at our hospital by injecting the same venom into mice. Our systemic investigations revealed that a cytokine storm—regulated by the NF‐κB p65 signaling pathway—is a critical factor in developing DJES following jellyfish venom exposure, even at low toxin doses. We show that dexamethasone, a broad‐spectrum anti‐inflammatory agent, effectively suppresses this cytokine storm, mitigates multi‐organ failure, and improves animal survival. These findings show that, besides the direct toxic effects of the venom, fatal outcomes of jellyfish stings are also caused by cytokine storms triggered by low doses of the toxins. Given the diversity of jellyfish species and venom compositions, this new understanding of DJES pathogenesis suggests that targeted treatments that modulate the host immune response may offer a more practical approach than the current practice of neutralizing toxins. Such treatments could also potentially be applied to other acute inflammatory conditions.

## 2 Results

### 2.1 DJES Mouse Model of Human Jellyfish Envenomation

**Figure 1:**
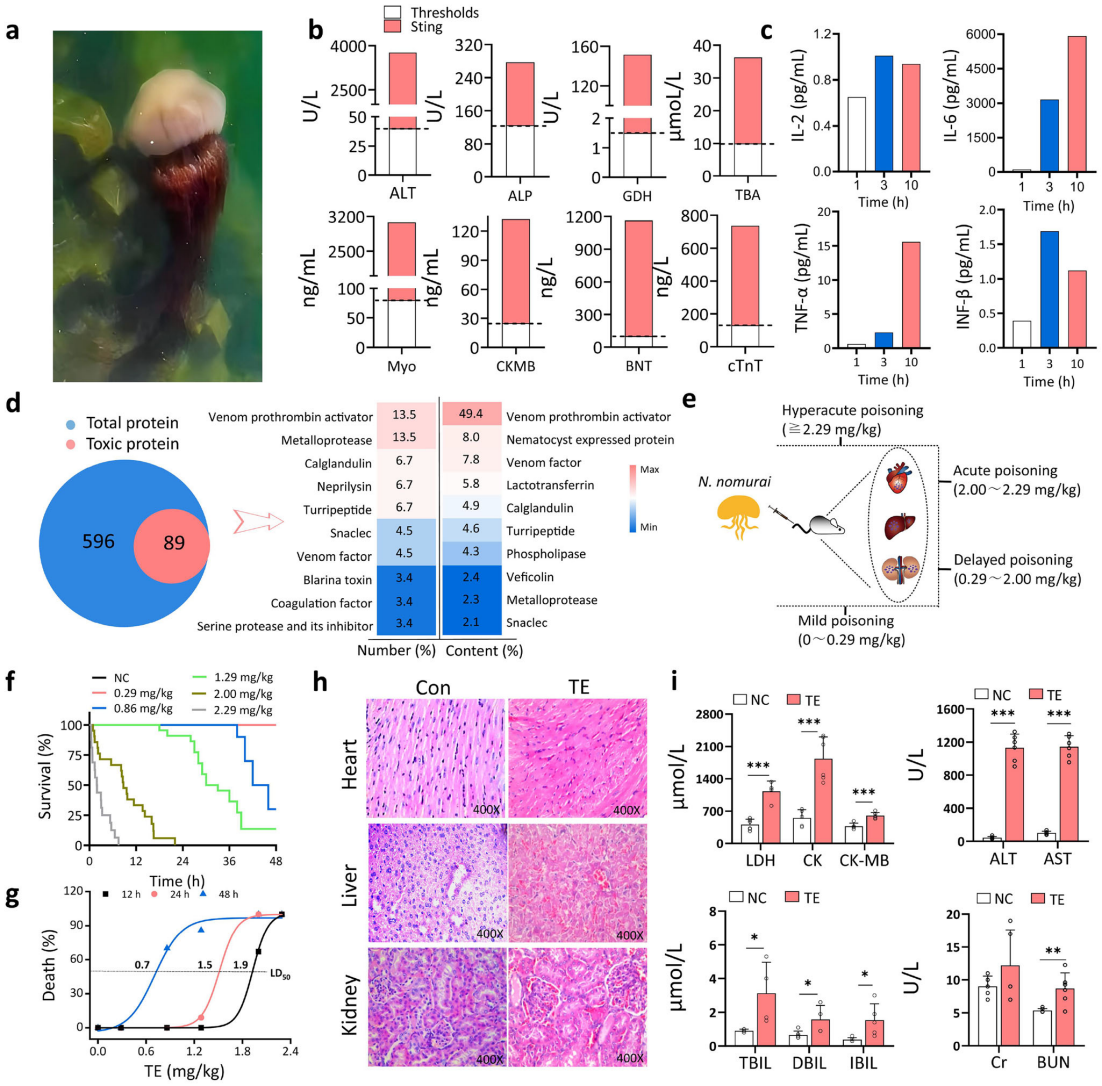
Establishing a DJES mouse model based on human jellyfish envenomation. a) Photographs of the N. nomurai jellyfish that fatally stung a patient and inspired this study. b) The stung patient's Serum biochemical indicators (ALT, ALP, GDH, TBA, Myo, CK‐MB, BNT, and cTnT) exceeded the acceptable threshold levels. c) Serum levels of inflammatory cytokines (IL‐2, IL‐6, TNF‐α, and IFN‐β) in the stung patient increased over time. d) Proteomic analysis of toxins' type and expression level in *N. nomurai* venom (obtained from its tentacles) shows that metalloproteinases dominate. e) Schematic diagram illustrating the establishment of the DJES mouse model via tail vein injection of venom. f) Survival rates of DJES mice injected with various concentrations of TE. The negative control mice received no venom. (n = 10 biologically independent samples). g) Mortality rates of DJES mice injected with different concentrations of TE at 12, 24, and 48 h. Over time, the dose causing 50% of animal death (LD50) (horizontal line) drops from 1.9 to 0.7 mg kg^−1^. h) Hematoxylin and eosin (H&E) staining of heart, liver, and kidney tissues six hours post‐envenomation shows damage in treated (TE) animals. Control (Con) animals received no venom. Magnification × 400. (n = 4 biologically independent samples). i) Higher serum biochemical indicators in DJES mice (TE) compared to control (NC) animals indicate significant damage to heart, liver, and kidney functions. Error bars are s.d. (n = 4–6 biologically independent samples). ^*^ *p* < 0.05; ^**^ *p* < 0.01; ^***^ *p* < 0.001 by Student's *t*‐ test.

We established a DJES mouse model based on a patient at our hospital who suffered fatal head and facial injuries from a *Nemopilema nomurai* jellyfish sting (**Figure** **1**a). The patient, who was admitted to the hospital 2–3 h after envenomation, displayed a tight chest and shortness of breath. Hematological analysis reveals damaged heart, liver, and kidney functions (Figure 1b). Elevated expression of IL‐2, IL‐6, TNF‐α, and IFN‐β indicated a severe acute inflammatory response that led to multi‐organ failure (Figure 1c). To replicate the patient's condition, we captured live *N. nomurai* jellyfish from the same area for further research on the DJES model and its potential mechanism elucidation.

Proteomic analysis showed the TE contained predominantly metalloproteinases and other toxic components such as staphylococcal toxins, coagulation factors, peroxiredoxins, and phospholipases, which commonly contribute to sting poisoning (Figure 1d and Table S1a,b, Supporting Information). Therefore, we injected various concentrations of the venom (tentacle extract, TE) into the tail vein of mice and monitored their condition and survival rates (Figure 1e).

As the venom concentration increased from 0 to 2.29 mg kg^−1^, the animals’ average time to death decreased from 48 to 12 h (Figure 1f). When the observation time extended from 12 to 48 h, the lethal dose to 50% of the animals (LD_50_) decreased from 1.9 to 0.7 mg kg^−1^ (Figure 1g). Like the patient, the treated animals showed weakened breathing, crouching, lethargy, convulsions, and eventually death. Histopathological studies performed 6 h after envenomation revealed edema and degeneration of myocardial fibers in the heart, loss of lobular structure with extensive hemorrhage and severe hepatocellular necrosis in the liver, and destruction of glomerular capillaries with fibrin microthrombi deposits and severe degenerative changes in the proximal tubules of the kidneys (Figure 1h). Additionally, biochemical indicators such as LDH, CK, ALT, AST, TBIL, BUN, and Cr in the DJES mice were significantly higher than the control animals that did not receive any venom (Figure 1i). These results indicate that we successfully simulated the patient's envenomation state and established a DJES mouse model that demonstrates damage to heart, liver, and kidney functions, and eventually death.

### 2.2 Jellyfish Envenomation Triggers Cytokine Storm in DJES Mice

**Figure 2:**
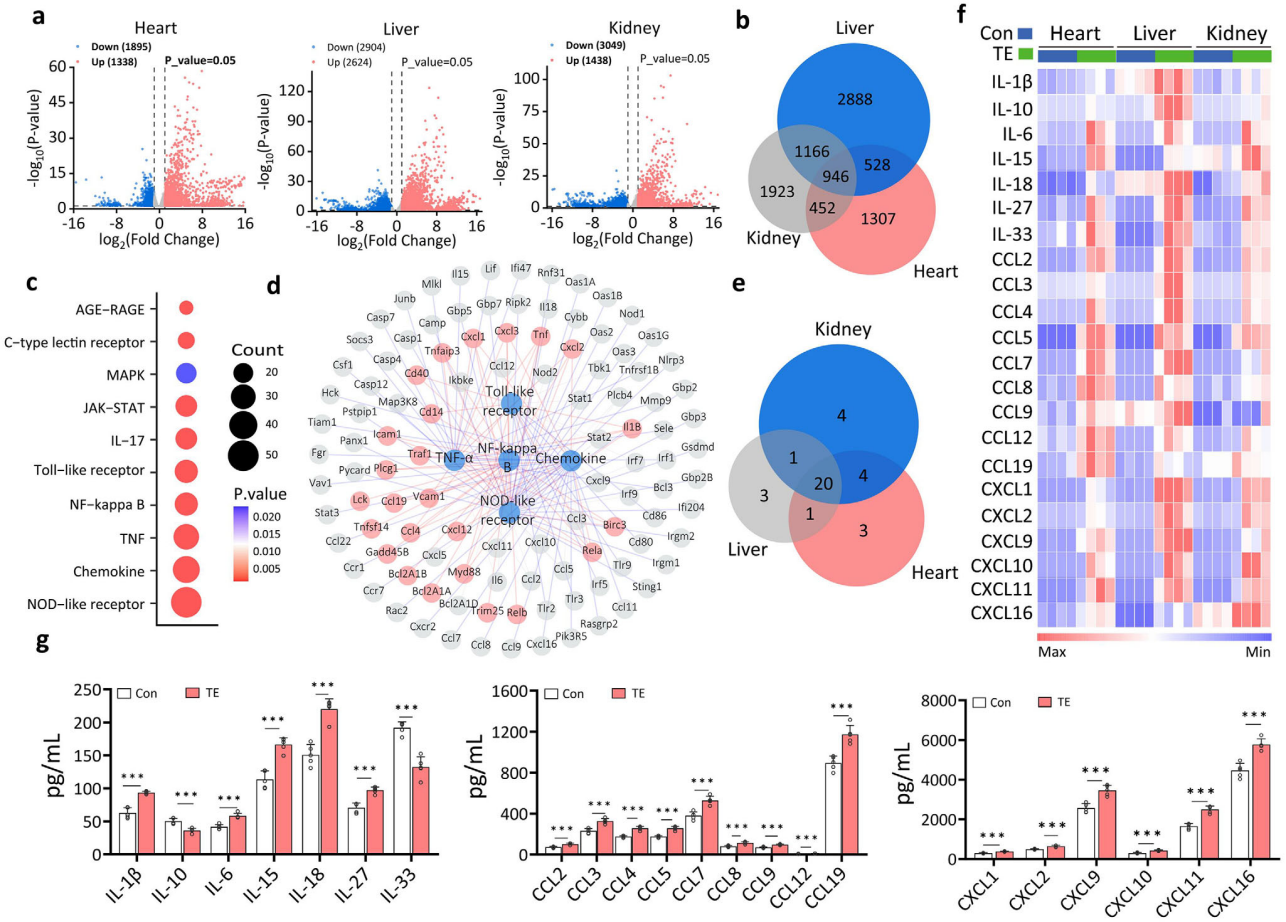
DJES mice experience a cytokine storm after jellyfish envenomation. a) Volcano plots of DEGs in the heart, liver, and kidneys of DJES mice (n = 4 biologically independent samples). Thresholds: |log_2_​FC| ≥ 1, p < 0.05. b) Venn diagram showing the 946 overlapping DEGs in the heart, liver, and kidneys. c) Co‐enriched KEGG pathways related to heart, liver, and kidney inflammation. d) Network analysis between the top five enriched KEGG pathways and their associated genes. Pathways (blue), DEGs in the NF‐κB pathway (red), and other genes (gray) are shown. The inner circle (red) contains genes enriched in two or more pathways; The outer circle contains genes enriched in one pathway. e) Venn diagram of the upregulated cytokines in the heart, liver, and kidneys. f) Heatmap of co‐upregulated cytokines in the heart, liver, and kidneys. g) ELISA measurements show serum levels of different cytokines in DJES mice (TE) are higher than in control (Con) animals. Error bars are s.d. (n = 3–5 biologically independent samples). ^*^ *p* < 0.05; ^**^ *p* < 0.01; ^***^ *p* < 0.001 by Student's *t*‐ test.

To investigate the mechanisms underlying multi‐organ failure in DJES, we performed transcriptomic analysis on the mice's hearts, liver, and kidneys. TE induced 3233, 5558, and 4487 differentially expressed genes (DEGs) in the heart, liver, and kidneys, respectively (**Figure** **2**a and Table S2a–c, Supporting Information). A total of 946 DEGs overlapped across all three organs (Figure 2b, Figure S1a,b, and Table S2d, Supporting Information).

Pathway enrichment analysis revealed that the top ten pathways were related to inflammation, including the NOD‐like receptor and chemokine signaling pathways (Figure 2c, Figure S1c,d, and Table S2e, Supporting Information). Notably, the NF‐κB signaling pathway was significantly perturbed (Figure 2d, Figure S1e, and Table S2f, Supporting Information). We identified 28, 25, and 29 upregulated cytokines in the heart, liver, and kidneys, respectively (Figure 2e and Figure S1f, Supporting Information). Among these, 20 cytokines (≈55.56%), including various ILs, CCLs, and CXCLs, were upregulated across all three organs (Figure 2f and Table S2g, Supporting Information). Quantitative RT‐PCR data showed a 95% match with the transcriptomic data for these cytokines (Figure S2a–c). ELISA measurements also show the serum levels of 20 cytokines, except for IL‐10 and IL‐33, increased significantly in DJES mice (Figure 2g). A compliance rate of over 90% indicates high reproducibility and consistency of the results. These findings show that jellyfish envenomation of DJES mice induces a cytokine storm and systemic inflammatory responses like those seen in the stung patient. This suggests that cytokine storms potentially cause multi‐organ failure in fatal jellyfish stings.

### 2.3 Macrophages are Responsible for Cytokine Storm in DJES

**Figure 3:**
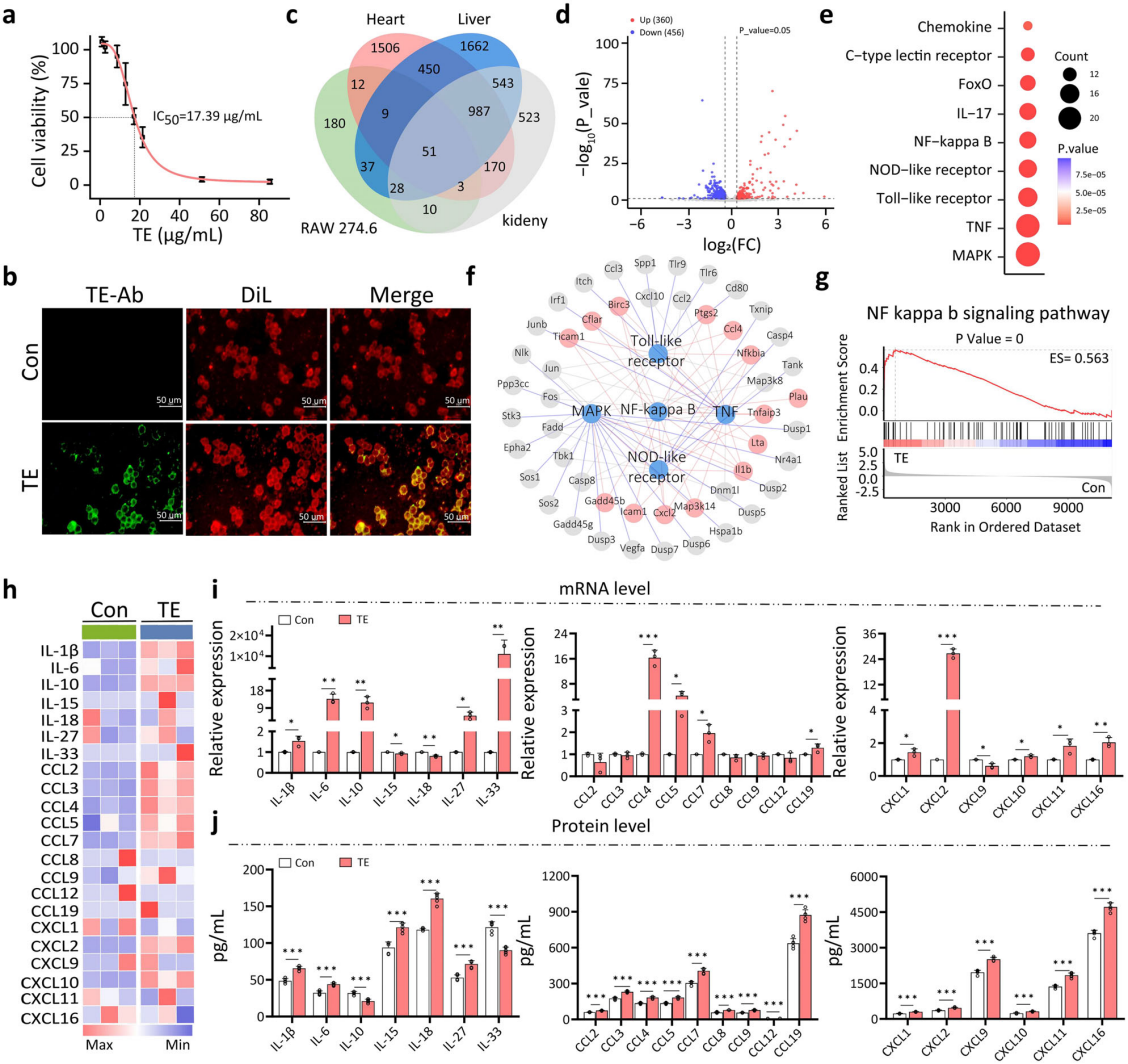
Upregulated cytokine expression in macrophages treated with TE. a) Cell viability of RAW 274.6 macrophages 6 h after TE administration. Error bars are s.d. (n = 6 biologically independent samples). b) Immunofluorescence staining of macrophages with a self‐made TE polyclonal antibody 2 h after TE administration. Cell membranes are stained with DiL (red), and bound TE is in green. Data are from one experiment and are representative of three independent experiments. c) Venn diagram shows DEGs induced in macrophages overlap with those in the heart, liver, and kidneys of TE‐treated DJES mice. d) Volcano plot of DEGs in macrophages (n = 3 biologically independent samples). Thresholds: ∣log_2_​FC∣ ≥ 0.378, *p* < 0.05. e, Bubble plot shows the top nine KEGG enriched pathways in treated macrophages are related to inflammation. f) Network analysis between the top five enriched KEGG pathways and their associated genes in macrophages. Pathways (blue), DEGs in the NF‐κB pathway (red), and other genes (gray) are shown. g) Gene set enrichment analysis (GSEA) plot of the NF‐κB pathway in macrophages displays an enrichment score of 0.563. h) Heatmap of 22 differentially expressed cytokine genes in TE‐treated (TE) and untreated (Con) macrophages. i) Quantitative RT‐PCR validation of cytokine gene expression in TE and Con macrophages. Error bars are s.d. (n = 3 biologically independent samples). j) ELISA measurements of cytokine levels in the cell supernatant. Error bars are s.d. (n = 5 biologically independent samples). ^*^ *p* < 0.05; ^**^ *p* < 0.01; ^***^ *p* < 0.001 by Student's *t*‐ test.

To understand how jellyfish envenomation induces the inflammatory cytokine storm, we treated RAW 274.6 macrophages to TE and examined their responses in vitro. Macrophages are a type of non‐specific immune cell known to be implicated in many forms of cytokine storms. CCK8 cytotoxicity assay shows the half maximal inhibitory concentration (IC_50_) of TE after 6 h is 17.39 µg mL^−1^ (**Figure** **3**a). Immunofluorescence studies revealed that TE bound to the macrophage membrane (Figure 3b and Figure S3a,b, Supporting Information).

Transcriptomic analysis of TE‐treated macrophages identified 51 DEGs that overlapped with those found in the heart, liver, and kidneys of TE‐treated DJES mice (Figure 3c,d, and Table S3a, Supporting Information). Like DJES mice, the top ten enriched pathways in the treated macrophages were also related to inflammation (Figure 3e and Table S3b, Supporting Information). Notably, NF‐κB p65 was activated, and the NF‐κB signaling pathway emerged as a central pathway with an enrichment score of 0.563 (Figure 3f,g, and Table S3c, Supporting Information).

Among the 22 cytokines analyzed, 15 (including CCL3, CCL4, and CXCL10) were upregulated in TE‐treated macrophages compared to untreated controls (Figure 3h and Table S3d, Supporting Information). Quantitative RT‐PCR confirmed that 14 cytokines, such as IL‐1β, IL‐6, and CCL4, were significantly increased (Figure 3i). At the protein level, 20 cytokines increased significantly except for the anti‐inflammatory cytokines, IL‐10 and IL‐33 (Figure 3j). These results are consistent with the cytokine profiles seen in DJES mice, and they indicate that TE induces macrophages to unleash a robust cytokine storm across various organs and tissues, which collectively leads to systemic inflammation.

### 2.4 NF‐κB p65 Mediates Jellyfish Venom‐Induced Cytokine Storms

**Figure 4:**
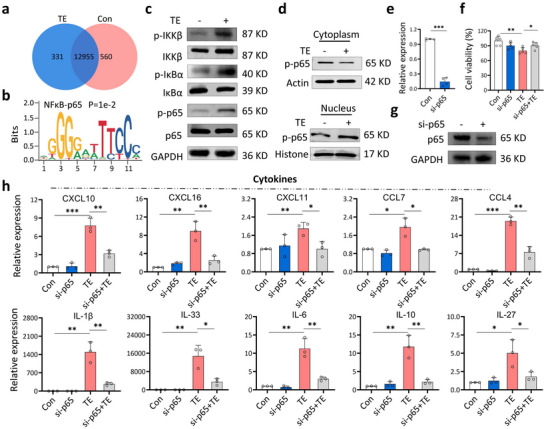
NF‐κB p65 is a key modulator of cytokine expression in macrophages. a) Venn diagram showing the genes identified by ChIP‐seq in TE‐treated (TE) and untreated (Con) macrophages. b) Motif sequence of NF‐κB p65 identified by ChIP‐seq. c) Western blot analysis shows NF‐κB p65 signaling pathway proteins and their phosphorylation, including IKKβ, IκBα, and p65, were higher in TE‐treated (+) than untreated (‐) macrophages. (n = 4–6 biologically independent samples). d) Western blot analysis of phosphorylated p65 in the cytoplasm and nucleus of TE‐treated and untreated macrophages. (n = 3 biologically independent samples). e) Quantitative RT‐PCR confirms efficient p65 knockdown using siRNA. Error bars are s.d. (n = 3 biologically independent samples). f) Viability of TE‐treated macrophages after p65 knockdown (si‐p65+TE) was significantly higher than TE‐treated cells that were not knocked down (TE). Error bars are s.d. (n = 5 biologically independent samples). g) Western blot confirms siRNA efficiently knocked down p65 in macrophages (n = 5 biologically independent samples). h) Cytokine levels in macrophages after p65 knockdown as measured by quantitative RT‐PCR. Error bars are s.d. (n = 3 biologically independent samples). ^*^ *p* < 0.05; ^**^ *p* < 0.01; ^***^ *p* < 0.001 by Student's *t‐* test.

Using ChIP‐seq analysis targeting the H3K4me3 histone that marks active chromatin regions associated with gene expression, we show NF‐κB signaling pathway mediates the cytokine storm. Out of 13846 genes bound to H3K4me3, 331 were specific to the TE‐treated macrophages (**Figures** **4**a and S4a–c and Table S4a,b, Supporting Information). KEGG pathway analysis enriched 25 signaling pathways associated with inflammation (Figure S4d and Table S4c, Supporting Information).

Genes related to the NF‐κB p65 signaling pathway included seven cytokines, such as IL‐6, IL‐10, CXCL16 (Figure S4b, Supporting Information), and the transcription factor p65 itself (Figure 4b). This suggests that NF‐κB p65 plays a key role in TE‐induced cytokine storms and systemic inflammatory responses. In macrophages, TE stimulation increased the expression and phosphorylation levels of IKKβ and IκBα, leading to the release and phosphorylation of p65 (Figure 4c and Figure S5a, Supporting Information). The phosphorylated p65 accumulated in the nucleus, where it functions as a transcription factor (Figure 4d and Figure S5b, Supporting Information).

Knocking down p65 expression in macrophages with siRNA (Figure 4e,f, and Figure S5c, Supporting Information) significantly increased cell viability after TE treatment (Figure 4g). The levels of at least 10 cytokines, including CXCL10, CXCL16, CCL7, CXCL11, CCL4, IL‐1β, IL‐33, IL‐6, IL‐10, and IL‐27, dropped significantly after the knockdown (Figure 4h). Collectively, these findings indicate that TE activates the NF‐κB p65 signaling pathway and that p65 is crucial to jellyfish venom‐induced inflammation.

### 2.5 Dexamethasone Inhibits p65 and Prevents Cytokine Storm

**Figure 5:**
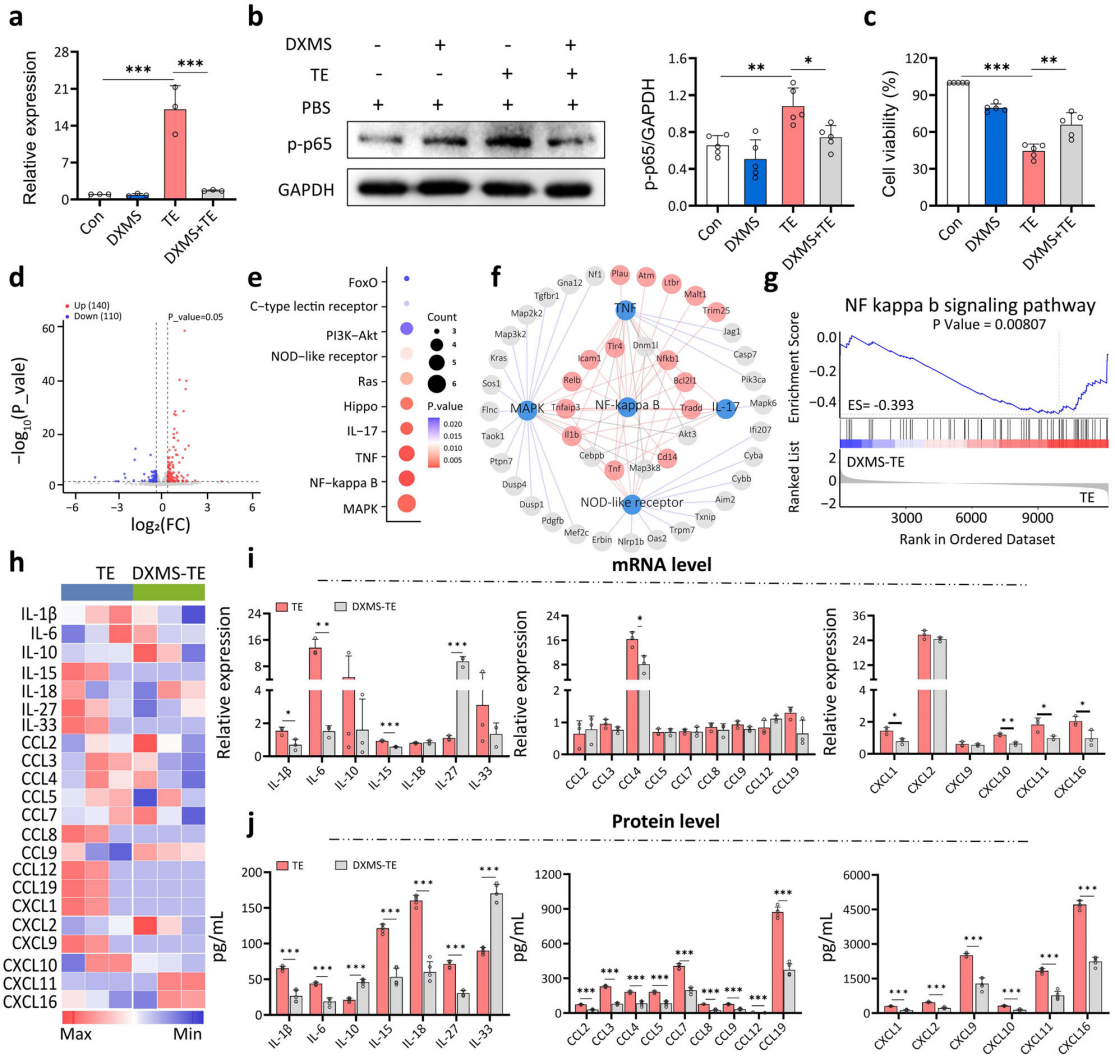
Dexamethasone inhibits cytokine expression in TE‐treated macrophages. a) Quantitative RT‐PCR confirmed p65 expression drops after DXMS intervention in TE‐treated macrophages. TE and DXMS concentrations were 10 µg mL^−1^ and 2 mm, respectively. Error bars are s.d. (n = 5 biologically independent samples). b) Western blot (left) and quantitative analysis (right) show DXMS suppressed p65 protein expression and phosphorylation in TE‐treated macrophages. Error bars are s.d. (n = 5 biologically independent samples). c) DXMS intervention improved the viability of TE‐treated macrophages. Error bars are s.d. (n = 5 biologically independent samples). d) Volcano plot of DEGs in macrophages. (n = 3 biologically independent samples). Thresholds: ∣log_2_​FC∣ ≥ 0.378, p < 0.05. e) KEGG pathway analysis shows the top 10 enriched pathways of DEGs between TE compared to the Con group. f) Network analysis between the top five enriched KEGG pathways and their associated genes after DXMS intervention. Pathways (blue), DEGs in the NF‐κB pathway (red), and other genes (gray) are shown. g) Gene set enrichment analysis (GSEA) plot of the NF‐κB pathway in TE‐treated macrophages shows an enrichment score of –0.393 after DXMS intervention. h) Heatmap of differentially expressed cytokines in macrophages treated with TE only (TE) or TE and DXMS (DXMS‐TE). i) Quantitative RT‐PCR validation of cytokine gene expression after DXMS intervention. Error bars are s.d. (n = 3 biologically independent samples). j) ELISA measurements show that besides IL‐10 and IL‐33, cytokine protein levels dropped after DXMS intervention. Error bars are s.d. (n = 5 biologically independent samples). ^*^ *p* < 0.05; ^**^ *p* < 0.01; ^***^ *p* < 0.001 by Student's *t*‐ test.

Dexamethasone (DXMS) is a well‐known anti‐inflammatory drug^[^ ^22^, ^23^ ^]^ that prevents the phosphorylation of p65.^[^ ^24^ ^]^ We investigated its efficacy in counteracting TE‐induced inflammation in macrophages. DXMS suppressed p65 gene expression and protein phosphorylation by 99% and 31%, respectively (**Figure** **5**a,b), and increased macrophage viability from 44.5% to 65.8% (Figure 5c). DXMS also inhibited the NF‐κB p65 signaling pathway with an enrichment score of – 0.393 and suppressed other inflammatory signaling pathways (Figure 5d–g, Figure S6a–d and Table S5a–f, Supporting Information).

Further, DXMS intervention in TE‐treated macrophages downregulated 14 out of 22 cytokine genes, including IL‐1β, CXCL10, and CCL4 (Figure 5h, Figure S6e, and Table S5g,h, Supporting Information). Quantitative RT‐PCR confirmed that expression levels of 9 cytokines (including CXCL1, IL‐6, and CXCL16) dropped, 11 cytokines showed no significant difference, and IL‐27 was upregulated (Figure 5i and Figure S7a, Supporting Information). At the protein level, DXMS effectively suppressed 20 cytokines, except for the anti‐inflammatory factors IL‐10 and IL‐33 (Figure 5j and Figure S7b, Supporting Information). Together, these results demonstrate that DXMS reduces cytokine expression and improves cell survival by inhibiting the inflammation modulator, p65.

### 2.6 Dexamethasone Improves Survival of DJES Mice

Encouraged by the in vitro results, we tested the anti‐inflammatory effects of DXMS in the DJES mouse model. The animals were injected with 2 mg kg^−1^ TE and various concentrations of DXMS, and their survival rate, organs, serum biochemical indicators, and cytokine expression were measured and compared to control animals that did not receive either DXMS, TE, or both.

**Figure 6:**
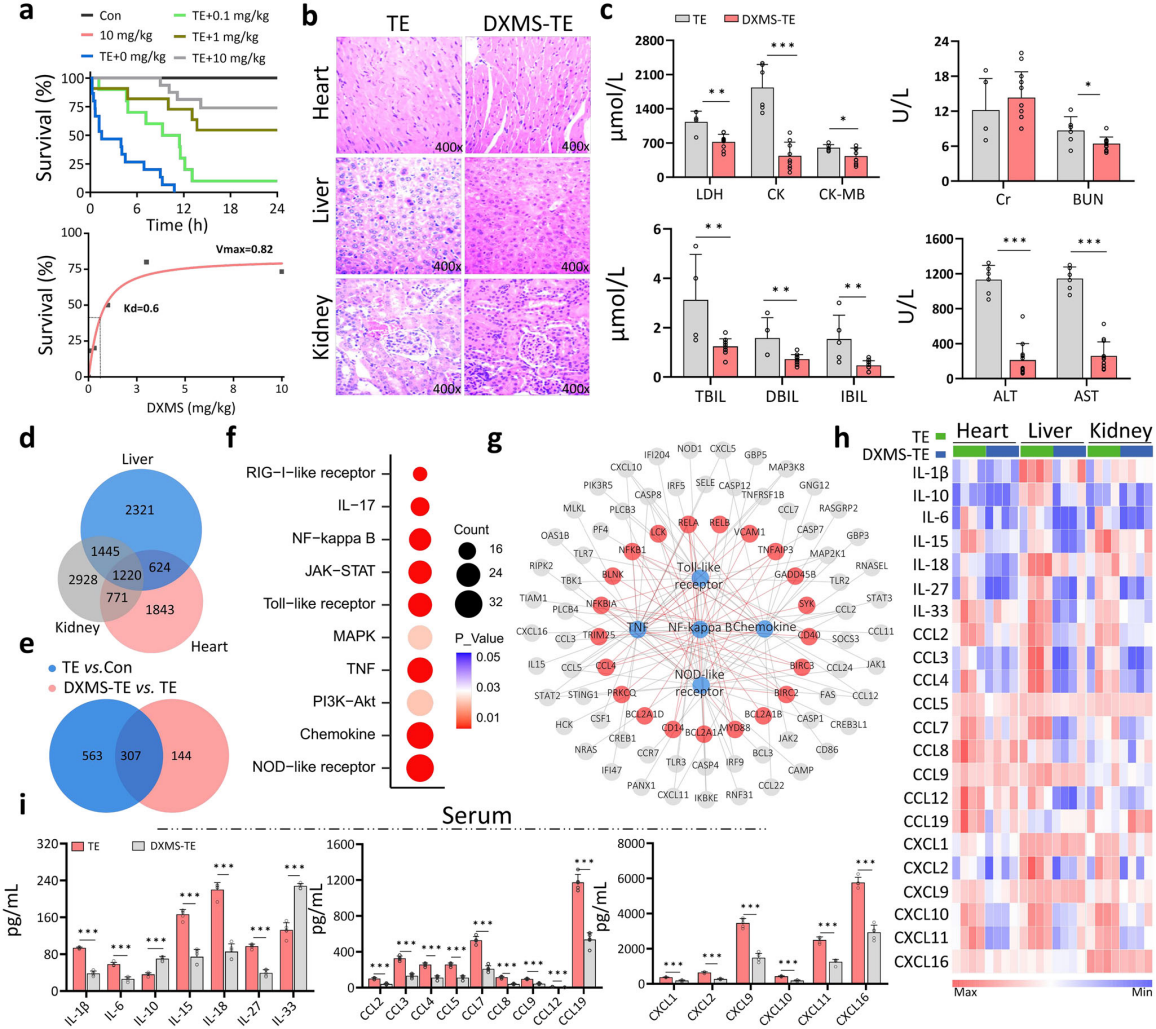
Dexamethasone inhibits cytokine storms and increases the survival of DJES mice. a) Survival rates of DJES mice improve with DXMS concentration. Animals were injected with 2 mg mL^−1^ TE. Con animals were treated with 1xPBS. (n = 10–15 biologically independent samples). b) H&E‐stained sections of the heart, liver, and kidney of DXMS‐treated DJES mice (DXMS‐TE) show less damage than untreated DJES animals (TE) (n = 4 biologically independent samples). c) Serum biochemical indicators reveal heart, liver, and kidney functions are restored after DXMS treatment. Error bars are s.d. (n = 4–6 biologically independent samples). d) Venn diagram of the DEGs in DXMS‐TE treated (DXMS‐TE) and TE‐treated (TE) macrophages. e) Venn diagram of all DEGs in the heart, liver, and kidneys of DXMS‐TE versus TE groups and TE versus Con groups. f) Top 10 KEGG pathways enriched by DEGs. g) Network analysis between the top five enriched KEGG pathways and their associated genes after DXMS intervention. Pathways (blue), DEGs in the NF‐κB pathway (red), and other genes (gray) are shown. h) Heatmap of cytokines in transcriptomes after DXMS intervention in DJES mice. (n = 4 biologically independent samples). i) ELISA results show cytokine protein levels after DXMS intervention in DJES mice. Error bars are s.d. (n = 5 biologically independent samples). ^*^ *p* < 0.05; ^**^ *p* < 0.01; ^***^ *p* < 0.001 by Student's *t*‐ test.

DXMS improved the survival rate of mice from 0 to 73% (**Figure** **6**a) and significantly reduced heart, liver, and kidney damage (Figure 6b). Serum biochemical indicators confirm that organ functions were restored after DXMS treatment (Figure 6c and Figure S8a,b, Supporting Information). Consistent with the in vitro findings, transcriptomic analysis of the heart, liver, and kidney tissues after DXMS intervention showed a significant decrease in the expression of ≈20 cytokines (Figure 6d–h and Figures S9,S10, Supporting Information). In agreement with the transcriptomic results (95%), quantitative RT‐PCR validation of five randomly selected cytokines further shows DXMS suppressed the inflammatory cytokines in these organs, except for IL‐27, which showed an opposite trend, and IL‐10 and IL‐15, which are anti‐inflammatory cytokines (Figure S11, Supporting Information). ELISA measurements also show that DXMS significantly suppressed the serum levels of 20 cytokines, except for IL‐10 and IL‐33 (Figure 6i and Figure S12, Supporting Information).

These results collectively demonstrate that DXMS improves the survival of DJES mice by inhibiting cytokine storms and preventing jellyfish envenomation‐induced damage in the heart, liver, and kidneys. Our findings indicate that cytokine storms play an essential role in DJES, and inhibiting excessive inflammation is crucial in mitigating organ damage.

## 3 Discussion

As seen in the clinical case, jellyfish envenomation can lead to severe inflammatory responses that culminate in multi‐organ failure and death. Here, we established a DJES mouse model and showed that jellyfish envenomation upregulates at least 20 cytokines (including TNF‐α, CXCL2, CXCL11, IL‐6, and CCL‐4) closely associated with inflammation. This pronounced cytokine response constitutes a cytokine storm – a severe inflammatory reaction known to cause tissue damage and organ failure in various conditions such as sepsis,^[^ ^25^, ^26^ ^]^ immunotherapy‐related adverse effects,^[^ ^27^, ^28^ ^]^ and viral infections like influenza and COVID‐19.^[^ ^29^, ^30^ ^]^ Discovering the link between jellyfish toxin and cytokine storm is significant because up until now, fatal outcomes of jellyfish stings have only been attributed to the direct toxic effects of venom components such as neurotoxins^[^ ^31^, ^32^, ^33^ ^]^ and cardiotoxins.^[^ ^34^, ^35^, ^36^ ^]^ This finding provides new evidence identifying the direct action of the venom as the primary cause of jellyfish stings. Not only that, we also show that even low doses of jellyfish toxins can trigger an exaggerated immune response that leads to potentially fatal DJES; this study will provide a new theoretical basis for the mechanism of jellyfish poisoning, especially for treating sting patients in clinical practice.

Our proteomic analysis of *N. nomurai* venom identified 685 proteins, including 89 toxin proteins such as metalloproteinases, phospholipases, and components that affect coagulation pathways. These toxins can initiate and amplify immune responses through several mechanisms. As foreign proteins, jellyfish toxins can directly stimulate immune cells like macrophages to release large amounts of cytokines.^[^ ^37^ ^]^ Specific venom components may act as pathogen‐associated molecular patterns (PAMPs) that activate innate immune receptors and downstream signaling pathways. Tissues and cells damaged by the toxins can release their intracellular content and Damage‐Associated Molecular Patterns (DAMPs), further stimulating the immune system to amplify cytokine production.^[^ ^16^ ^]^ Pro‐inflammatory cytokines such as IL‐1β and IL‐6 can create positive feedback loops that continuously recruit and activate additional immune cells to release more cytokines.^[^ ^38^, ^39^ ^]^ The delayed onset of DJES also provides a prolonged time window during which cytokine levels can accumulate, leading to sustained inflammation and organ damage.^[^ ^40^, ^41^ ^]^

We further show that the NF‐κB p65 signaling pathway mediates the cytokine storm induced by jellyfish venom. ChIP‐seq analysis identified p65 as a molecular switch regulating multiple cytokines.^[^ ^42^ ^]^ Knockdown of p65 expression in our study significantly reduced cytokine levels^[^ ^43^, ^44^ ^]^ and improved macrophage viability.^[^ ^45^ ^]^ Moreover, dexamethasone, a known inhibitor of the NF‐κB pathway^[^ ^46^ ^]^ that can effectively suppress cytokine storms^[^ ^47^ ^]^ and mitigate organ damage,^[^ ^48^ ^]^ increased the survival rates of the DJES mice in our study. These findings align with observations in sepsis^[^ ^49^ ^]^ and viral infections,^[^ ^50^ ^]^ where NF‐κB signaling is a crucial mediator of inflammatory responses.

Identifying cytokine storm as a critical mediator of DJES and the role of the NF‐kB p65 pathway in these responses offer valuable insights into the molecular mechanisms underlying toxin‐induced inflammation in general. Our study reveals how immune responses can become dysregulated following envenomation and provides a model for studying cytokine storms in other acute inflammatory conditions. Clinically, these findings suggest that targeting the NF‐κB p65 signaling pathway could mitigate the harmful effects of jellyfish stings. The effectiveness of dexamethasone at suppressing the cytokine storm and improving survival rates of DJES mice suggests anti‐inflammatory therapies could be repurposed or developed for treating jellyfish envenomation. This approach represents a strategic shift from solely focusing on neutralizing toxins to modulating the host immune response, which may be more practical given the diversity of jellyfish species and venom compositions.

While the present mouse model replicated the critical aspects of human DJES, it does not capture the full complexity of the human immune response. Pathways other than NF‐κB p65 focused here may be at play and warrant further investigation. Identifying additional inflammatory signaling pathways and immune cell interactions could pinpoint new therapeutic targets. Because the venom composition of the jellyfish can vary across species and environmental conditions,^[^ ^51^, ^52^ ^]^ future studies will need to examine other jellyfish species before the results can be safely generalized. Anti‐inflammatory drugs are effective in the treatment of jellyfish stings,^[^ ^16^, ^53^ ^]^ but there are less reports on preventive drugs. In current study, treating jellyfish stings with anti‐inflammatory drugs (DXMS) is promising and effective. Integrating anti‐inflammatory strategies with existing treatment protocols could improve patient outcomes and provide a comprehensive approach to managing jellyfish envenomation and related inflammatory disorders.

## 4 Experimental Section

### Patient and Sample

Serum samples were collected from a patient stung by jellyfish. Blood biochemical indicators and cytokines were quantitatively assessed. This research was conducted with approval from the Medical Ethics and Human Clinical Trial Committee.

### Cytokine Secretion Assay

The concentrations of human serum cytokines in the serum sample from the stung patient were determined by an ELISA kit (R&D Systems) following the manufacturer's protocol as previously described.

### Jellyfish *N. nomurai* Collection and Toxin Extraction

Jellyfish *N. nomurai* were captured from the sea area and transported in seawater to the laboratory. Fresh jellyfish tentacles were separated from tissues and aliquoted into 50 mL Rnase‐free tubes. All samples were stored at ‐80 °C before use.

Jellyfish toxin (tentacle extract) was extracted as the following instructions. Jellyfish tentacles were continuously stirred at 4 °C for 72 h and then filtered. The filtrate was centrifuged at 4 °C and 10,000 g for 15 min. The supernatant was taken and dialyzed overnight in 1×PBS before use.

### Preparation of Polyclonal Antibodies

The polyclonal antibody against jellyfish toxins was prepared with the help of HUABIO Biotechnology Co., Ltd (Zhejiang, China). The antibody's valence was evaluated using western blotting (WB) analysis (Figure S3, Supporting Information).

### Animal Model of DJES and Survival Analysis

ICR mice (6–8 weeks, provided by the Laboratory Animal Center of Naval Medical University, Shanghai) were used to establish the jellyfish‐stinging model with varying doses of tentacle extract (TE) or DXMS. Briefly, ICR mice were injected with jellyfish toxin (0–2.29 mg kg^−1^) through the tail vein and were subsequently observed continuously for 48 h. The median lethal dose (LD_50_) was calculated. For the DXMS intervention test, mice were divided into four groups: the PBS group, the DXMS group, the TE group, and the TE‐DXMS group. Tissue samples from the heart, liver, and kidney of each group were fixed in formalin for histopathology analysis as previously described.^[^ ^54^ ^]^ Orbital blood samples were collected to analyze the biochemical indicators using the supernatant obtained after centrifugation at 2,000 g for 10 min. This study followed the National Institutes of Health Guide for the Care and Use of Laboratory Animals. It was approved by the Laboratory Animal Ethics Committee of Naval Medical University (Second Military Medical University).

### Histopathological Analysis and Blood Biochemical Analysis

Tissue samples from the heart, liver, and kidney were fixed in 10% neutral formaldehyde, washed with PBS, and eluted with alcohol gradient elution. Following paraffin embedding, the samples were cut into slices. The slices were then dewaxed with xylene, eluted with alcohol gradient elution, stained with hematoxylin‐eosin (H&E), and subjected to alcohol gradient elution once again. Subsequently, they were clarified with xylene and mounted by adding neutral balata. Pathological changes were observed under a light microscope.

Orbital blood samples were collected from ICR mice 6–8 h after TE injection. The supernatant obtained after centrifugation at 2,000 g for 10 min was utilized to detect blood biochemical indexes, including heart markers LDH, CK, and CK‐MB; liver markers ALT and AST; and kidney markers Cr and BUN.

### Cell Culture

RAW264.7 cells cultured in DMEM supplemented with 10% FBS under a 95% humidified atmosphere containing 5% CO_2_ at 37 °C, as previously described^[^ ^55^ ^]^ were used to establish the cell model with varying doses of TE or DXMS. Briefly, RAW264.7 cells were incubated with jellyfish toxin and were subsequently observed continuously for 6 h. For the si‐p65 or DXMS intervention test, the cells were divided into four groups: the PBS group, TE group, si‐p65 or DXMS group, and TE‐DXMS or TE‐si‐p65 group (cells initially incubated with si‐p65 or DXMS for 1 h followed by the addition of TE for further incubation). After incubation, the cells were washed twice with PBS, lysed with SDS lysis buffer containing PMSF, protease inhibitor, and phosphatase inhibitor for 1–2 s, then transferred into a centrifuge tube. The cells were disrupted using a 65 W cell sonicator for 3 s, followed by incubation on ice for 5 min and centrifugation at 14,000 g at 4 °C for 5 min. The supernatant was collected, and the protein concentration was determined using a standard BCA assay kit (Beyotime).

### siRNA Transfection

Small interfering RNA (RNAi) against mouse p65 and corresponding scramble siRNA were synthesized by Ribobio (Guangzhou, China). As previously described, macrophages were transfected with siRNA (50 nm) via Lipofectamine 3000 (Invitrogen) according to the manufacturer's protocols before incubation with indicated agents, and harvested for further investigation.

### CCK8 Assay

Cell viability was assessed using the Cell Counting Kit (CCK8). RAW264.7 cells were seeded in 96‐well culture plates at 1×10^4^ cells mL^−1^ density. After 24 h of incubation, the cells were exposed to varying concentrations of TE (0–800 µg mL^−1^, n = 6) for 6 h. Subsequently, the supernatant was aspirated and replaced with 90 µL of DMEM (BasalMedia) and 10 µL of CCK8 reagent in each well, followed by incubation for an additional 4 h at 37 °C. After that, the absorbance was measured at 450 nm using a microplate reader (BioTek). The cell survival rate was calculated using the formula: survival% = 100 × (OD_TE_ – OD_background_)/(OD_negative_ – OD_background_).

### Immunofluorescence Staining

RAW264.7 cells were cultured in 24‐well plates at a density of 1×10^5^ cells mL^−1^ for 12 h. Cells in the experimental group were incubated with jellyfish toxin for 2 h followed by three washes with 500 µL of pre‐chilled PBS for 5 min each. Subsequently, the cells were fixed with 200 µL of immunostaining fixative (Beyotime) for 15 min and then washed three times for 5 min each. Following this, 200 µL of immunostaining permeabilization solution (Beyotime) was added to cover the cells for 10 min, followed by three washes for 5 min each. The cells were then blocked with 200 µL of QuickBlock Immunostaining Confinement Solution (Beyotime) for 60 min and incubated overnight at 4 °C with jellyfish toxin polyclonal antibody. Subsequently, the jellyfish polyclonal antibody was removed, and the cells were washed three times with PBS for 5 min each. Hereafter, 200 µL of FITC Goat Anti‐Rabbit IgG (ABclonal) was added and incubated for 2 h and then washed three times with PBS for 5 min. The cell membrane was labeled by adding 200 µL of Dil staining solution (Beyotime), and the cell nucleus was labeled with 100 µL of DAPI (Beyotime) for 5 min at room temperature. Finally, the fluorescence microscope adjusted to 400× magnification was used to observe the results of each group as previously described.^[^ ^56^ ^]^

### Western blot Analysis

The expression levels of molecules were measured by Western blot. After being prepared and treated with TE, DXMS, or si‐p65, the total cell protein lysates of RAW264.7 were defrosted on ice for 30 min before use. The protein concentration was quantified using the standard BCA assay kit (Beyotime). SDS‐PAGE (4–15%, BioRad) was performed at 200 V for 30–40 min. Gels were transferred onto PVDF membranes (OIMG) at 100 V for 1 h at 4 °C. Membranes were then blocked with Protein Free Rapid Blocking Buffer (1×) (Yamei) for 1 h at room temperature.

NF‐κB p65 (D14E12) XP Rabbit mAb (CST, #8242), Phospho‐NF‐κB p65 (Ser536) (93H1) Rabbit mAb (CST, #3033), IKKβ (D30C6) Rabbit mAb (CST, #8943), Phospho‐IKKα/β (Ser176/180) (16A6) Rabbit mAb (CST, #2697), IκBα (44D4) Rabbit mAb (CST, #4812), Phospho‐IκBα (Ser32) (14D4) Rabbit mAb (CST, #2859), GAPDH (14C10) Rabbit mAb (CST, #2118), β‐Actin (13E5) Rabbit mAb (CST, #4970), Histone H3 (D1H2) XP Rabbit mAb (CST, #4499) were added to 5 mL of protein dilution (Beyotime) according to the instructions. Then, gels were placed in a sealed incubation box and incubated overnight with the membrane at 4 °C. Next, the membrane was washed 3 times with TBST and incubated with diluted secondary antibodies containing HRP‐conjugated Goat anti‐Rabbit IgG (ABclonal). Finally, the membrane was washed 3 times with TBST and visualized using a CCD camera from the Odyssey Fc imaging system (Licor) by running Precision Plus Protein Dual Color Protein Ladder (Bio‐Rad) and scanning different protein imprints at 700 nm/800 nm on the same system. Intensities of the band were quantified by Image J as previously described.^[^ ^57^ ^]^

### RNA Extraction and Transcriptome Sequencing

RNA was extracted from RAW246.7 cells or animal samples in the PBS, DXMS, TE, and DXMS‐TE groups using an RNAfast200 (Shanghai Feijie) as previously described. Briefly, RNA purity and quantity were analyzed with RNA 6000 Nano LabChip Kit (Agilent) and Bioanalyzer 2100.^[^ ^58^ ^]^ The library was prepared using the Sense Total RNA seq Library Prep Kit (Lexogen), and 75 nucleotides were sequenced using NextSeq500 (Illumina).^[^ ^59^, ^60^ ^]^ All assembled unigenes were annotated with a threshold of e‐value <0.00001. Databases such as Gene Ontology (GO) (http://www.geneontology.org) and Kyoto Encyclopedia of Genes and Genomes (KEGG) (http://www.genome.jp/kegg/) were annotated, respectively.

### qRT‐PCR Determination

Reverse transcription was performed using reverse transcription kits (Takara) to transcribe 1 µg of total RNA into cDNA. qRT‐PCR was performed using SYBR Prime Scrip RT PCR kits (Takara) on the 7300 plus Real‐Time PCR system as following conditions: denaturation at 95 °C for 5 min; denaturation at 94 °C for 20 s; annealing at 60 °C for 20 s; extension at 72 °C for 30 s; the last 3 stages consist of 40 cycles. The primer sequences of 23 cytokines were described in Table S8 (Supporting Information) and synthesized by Ribobio Biotechnology Co., Ltd.

### ELISA

The serum of animals or cell supernatant in PBS, DXMS, TE, and DXMS‐TE groups were collected respectively and used for determination of the expression levels of 23 cytokines (CXCL11, CCL7, IL‐33, CCL5, CXCL10, CCL4, IL‐15, CCL3, IL‐18, CCL2, CCL19, IL‐27, CCL9, CXCL1, IL‐6, CXCL2, CXCL9, TNF‐α, CCL12, CXCL16, CCL8, IL‐1β, IL‐10 and IL‐17) with five replicates.

### ChIP Analysis

The transcription factors binding to histones were analyzed by ChIP assay using the kit (CST, 56383S). Briefly, the RAW246.7 cells (1×10^6^) were cracked, ultrasonically treated for 48 cycles with a 20 s pause, and the supernatant was collected by centrifugation. The magnetic beads were then coupled with the target protein antibody (or IgG). In the IP group, the magnetic beads coupled with the antibody were incubated with the sample to bind the antibody and the target protein. H3K4me3 (CST, C42DB) antibodies were used to immune‐precipitate the H3K4me3‐chromatin complex. Then, magnetic beads were used to separate and wash the target protein to enrich it and its binding DNA. Anti‐IgG (Santa Cruz) was used as a blank control group to exclude the influence of other factors, as previously described.^[^ ^61^ ^]^

### Statistical Analysis

All data are presented as means ± standard deviation (SD). The statistical analysis was performed with a one‐way analysis of variance (ANOVA) and a Two‐tailed Student's test. The results were deemed statistically significant when *p* < 0.05.

## Conflict of Interest

The authors declare no conflict of interest.

## Author Contributions

Y.W., Y.W. and F.Y. contributed equally to this work. Conceptualization and methodology, Y.W. and L.X.; investigation, Y.W., Y.W., F.Y., J.C., X.G., Q.S., J.Z. and C.L.; transcriptomic analysis, Y.W., Y.W. and F.Y.; model construction and analysis, C.L. and J.L.; gene expression verification, X.H., Y.W. and H.L.; formal analysis, J.Z., L.C., B.Y. and L.X.; resources, B.Y. and L.X.; writing—original draft, L.C. and B.Y.; writing—review and editing, all authors; funding acquisition and supervision, L.X.

## Supporting information



Supporting Information

## Data Availability

The data that support the findings of this study are available from the corresponding authors upon reasonable request.
