# Supplementary material for: Cytokine Storm Induction Linked to Multi‐Organ Failure in Fatal Jellyfish Stings
Source: Adv Sci (Weinh). 2025 Jul 13;12(37):e01104. doi: 10.1002/advs.202501104 (PMC12499386; doi:10.1002/advs.202501104)
Supplement: Supplementary file 1 — Supporting Information [file ADVS-12-e01104-s001.pdf]

ADVANCED SCIENCE
Open Access

Supporting Information

for *Adv. Sci.*, DOI 10.1002/advs.202501104

Cytokine Storm Induction Linked to Multi-Organ Failure in Fatal Jellyfish Stings

Yichao Wang, Yi Wang, Fengling Yang, Jingbo Chen, Xiaoyu Geng, Qing Sun, Jinyu Zhang, Chang Liu, Jie Lv, Xiaochuan Hou, Yongfang Wang, Huiyan Lin, Jing Zhang, Lingxin Chen, Bing Yan* and Liang Xiao**

Research Article

Cytokine Storm Induction Linked to Multi-Organ Failure in Fatal Jellyfish Stings

Yichao Wang[1,2,#], Yi Wang[1,3,#], Fengling Yang[1,8,9,#], Jingbo Chen[1], Xiaoyu Geng[1], Qing Sun[4], Jinyu Zhang[1], Chang Liu[1,3], Jie Lv[1], Xiaochuan Hou[1], Yongfang Wang[1], Huiyan Lin[5], Jing Zhang[3], Lingxin Chen[6,*], Bing Yan[7,*], Liang Xiao[1,8,9,*]

[#] These authors contributed equally [*] These authors jointly supervised this work

[1] Faculty of Naval Medicine, Naval Medical University, Shanghai, 200433, China; wyc0708@smmu.edu.cn (Y.W.); wy0815@smmu.edu.cn (Y.W.); yfl1205@smmu.edu.cn (F.Y.); jimmychen777@smmu.edu.cn (J.C.); gxy0827@smmu.edu.cn (X.G.); zjy1024@smmu.edu.cn (J.Z.); lc0830@smmu.edu.cn (C.L.); lj0419@smmu.edu.cn (J.L.); hxc0214@smmu.edu.cn (H.X.) wangyongfang92@smmu.edu.cn (Y.W.); xiaolian@smmu.edu.cn (L.X.)

[2] Department of Clinical Laboratory, The First People's Hospital of Xianyang, Xianyang, 712000, Shannxi, China. wyc0708@smmu.edu.cn (Y.W.)

[3] College of Traditional Chinese Medicine, Jilin Agricultural University, Changchun, 130118, Jilin, China; wyi0815@hotmail.com (Y.W.); lc0830@smmu.edu.cn (C.L.); zhjing0701@163.com (J.Z)

[4] Department of Military and Special Medicine, No.971 Hospital of the PLA Navy, 266071, Qingdao, China (Q.S.); dr.qingsun@smmu.edu.cn

[5] Department of Intensive Care Medicine, No.971 Hospital of the PLA Navy, 266071, Qingdao, China; lihyicu@126.com

[6] CAS Key Laboratory of Coastal Environmental Processes and Ecological Remediation, Research Center for Coastal Environmental Engineering and Technology, Yantai Institute of Coastal Zone Research, Chinese Academy of Sciences, 264003, Yantai, China; lxchen@yic.ac.cn (L.C.)

[7] Institute of Environmental Research at the Greater Bay Area, Key Laboratory for Water Quality and Conservation of the Pearl River Delta, Ministry of Education, Guangzhou University, Guangzhou, 510006, China; drbingyan@gzhu.edu.cn (B.Y.)

[8] Key Laboratory of Biological Defense, Ministry of Education, Second Military Medical University, Shanghai, 200433, China; yfl1205@smmu.edu.cn (F.Y.); xiaolian@smmu.edu.cn (L.X.)

[9] Shanghai Key Laboratory of Medical Bioprotection, Second Military Medical University, Shanghai, 200433, China; yfl1205@smmu.edu.cn (F.Y.); xiaolian@smmu.edu.cn (L.X.)

[*]Corresponding authors: E-mail address: hormat830713@hotmail.com (L.X.); drbingyan@gzhu.edu.cn (B.Y.) lxchen@yic.ac.cn (L.C.).

Extended Data

Extended Data Figure List:

Extended Data Figure 1 | Differential gene expression reveals the molecular effect of multi-organs in DJES mice.
Extended Data Figure 2 | Quantitative RT-PCR validation of cytokine genes randomly selected in multi-organs.
Extended Data Figure 3 | Customized polyclonal antibody against jellyfish venom.
Extended Data Figure 4 | ChIP-seq analysis with TE treatment in macrophages.
Extended Data Figure 5 | Quantitative analysis of WB experiment.
Extended Data Figure 6|The physiological impact of Dexamethasone on macrophages.
Extended Data Figure 7|The effect of dexamethasone on cytokine expression in macrophages.
Extended Data Figure 8 | The effect of dexamethasone on physical effect of mice.
Extended Data Figure 9 | The effect of dexamethasone on the heart, liver and kidney in mice.
Extended Data Figure 10 | The effect of dexamethasone on cytokine storm in mice.
Extended Data Figure 11 | The effect of dexamethasone on cytokine expression on Heart, Liver and Kidneys.
Extended Data Figure 12 | ELISA results showed cytokine protein levels increased in DXMS-treated (DXMS) than Control (Con) group.

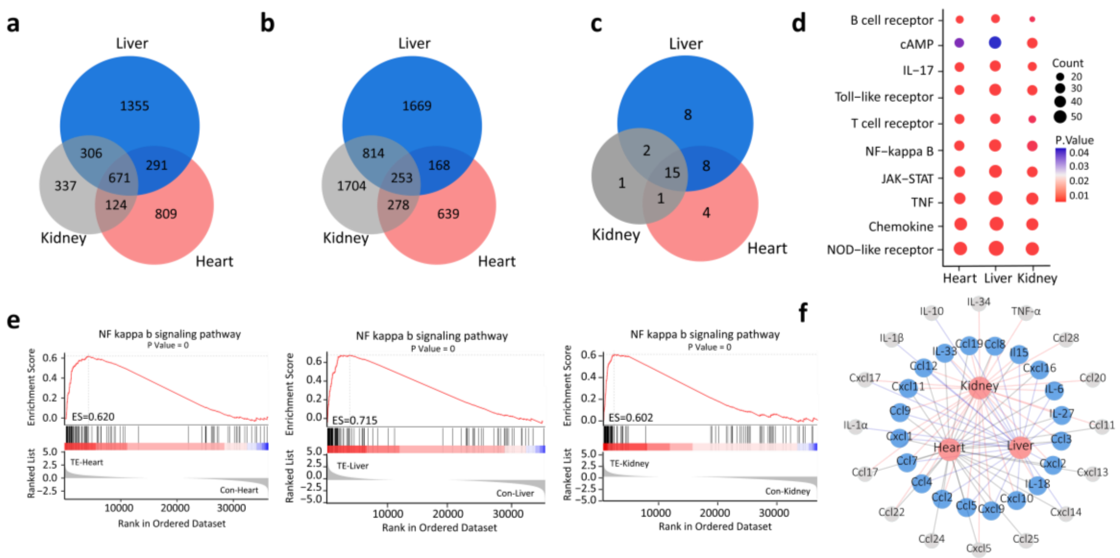

Extended Data Figure 1 | Differential gene expression reveals the molecular effect of multi-organs in DJES mice. a, Venn diagram showing the 671 numbers of overlapping up-regulated genes in the heart, liver, and kidneys. **b,** Venn diagram showing the 253 numbers of overlapping down-regulated genes in the heart, liver, and kidneys. **c,** Venn diagram showing the 15 overlapping KEGG pathway in heart, liver, and kidneys. **d,** The top 10 KEGG pathways co-enriched in the heart, liver, and kidneys. **e,** GSEA analysis of the upregulated NF-κB signaling pathway in the heart, liver and kidneys. **f,** The network image shows upregulated cytokines across the heart, liver, and kidneys. The inner circle (blue) represents co-upregulated cytokines in Heart, liver and kidneys, the outer circle represents genes enriched in one or two organs (n = 4 biologically independent samples).

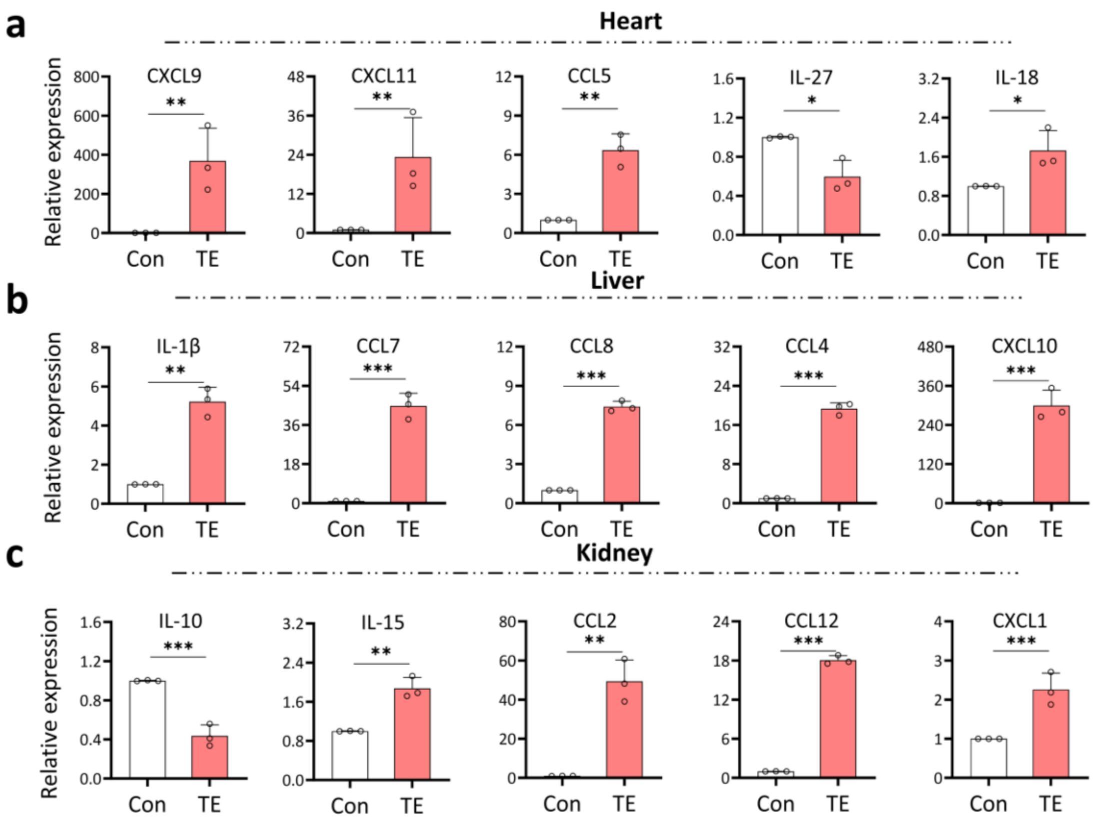

Extended Data Figure 2 | Quantitative RT-PCR validation of cytokine genes randomly selected in multi-organs. a, Five genes (CXCL9, CXCL11, CCL5, IL-27 and IL-18) up-regulated in heart were verified. **b,** Five genes (IL-1β, CCL7, CCL8, CCL4 and CXCL10) up-regulated in liver were verified. **c,** Five genes (IL-10, IL-15, CCL2, CCL12 and CXCL1) up-regulated in kidney were verified. Error bars represent s.d. (n = 3 biologically independent samples). *$p < 0.05$; **$p < 0.01$; ***$p < 0.001$ by Student's t test.

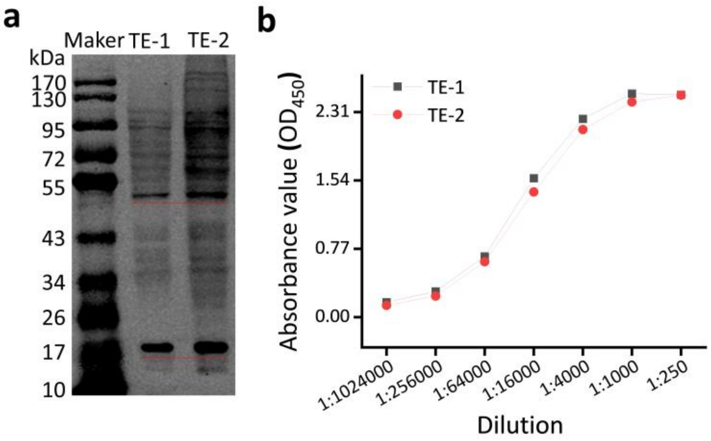

Extended Data Figure 3 | Customized polyclonal antibody against jellyfish venom. **a**, Western blot test of TE polyclonal antibody binding to TE proteins. **b**, The efficacy of TE polyclonal antibody from 1: 1024000 to 1: 250.

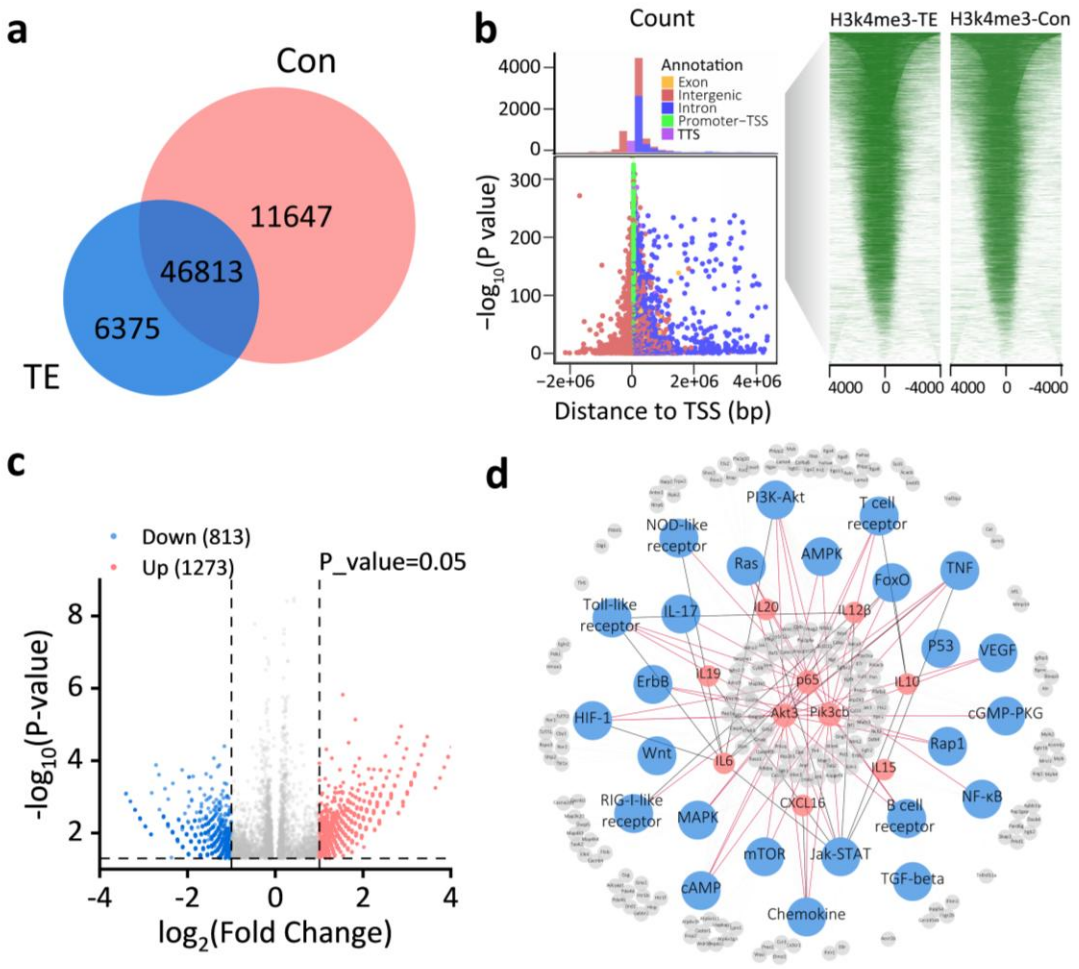

Extended Data Figure 4 | ChIP-seq analysis with TE treatment in macrophages. a, Venn result of gene fragments enriched by ChIP between Con and TE groups. **b**, The distribution and quantity of different types of gene fragments enriched by ChIP-seq near TSS. **c**, Volcanic map of differentially enriched gene fragments between TE group and Con group. $|\log_2 FC| \geq 1$, $p < 0.05$. **d,** KEGG pathway analysis showing enriched inflammatory pathways and related gene networks from ChIP-seq data. Blue circles represent pathways; The inner red circles represent important transcription factors; The outer red circles represent cytokines; The gray circles represent other genes.

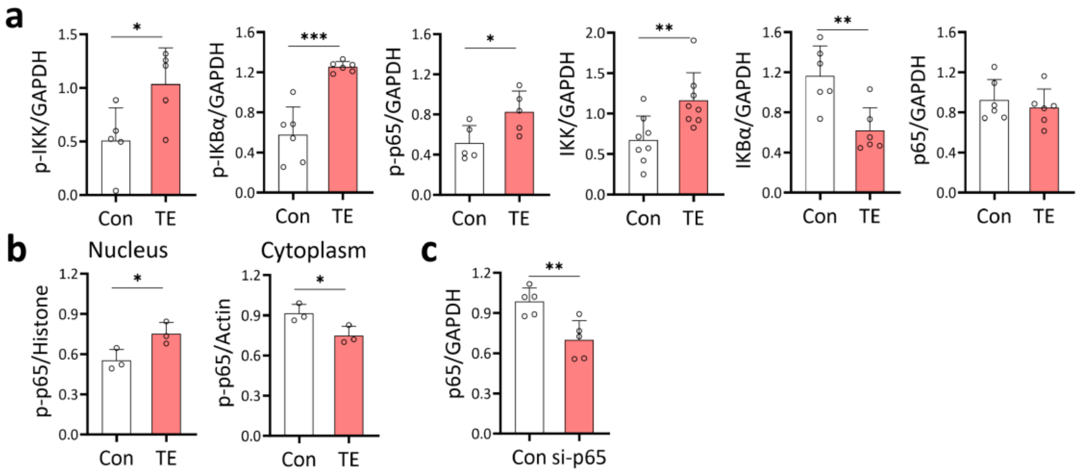

Extended Data Figure 5 | Quantitative analysis of WB experiment. a, Relative expression analysis of p-IKK, p-IKBα, p-p65, IKK, IKBα, and p65 at protein level in total protein samples. Error bars are s.d. (n = 4–8 biologically independent samples). **b**, WB quantitative analysis showed an increase in p-p65 protein levels in the cytoplasm and nucleus. Error bars are s.d. (n = 9 biologically independent samples). **c,** WB quantitative analysis showed an increase in p-p65 protein levels in TE while decreased after DXMS intervention. Error bars are s.d. (n = 5 biologically independent samples). Error bars are s.d. (n = 5 biologically independent samples). *p < 0.05; **p < 0.01; ***p < 0.001 by Student's t test.

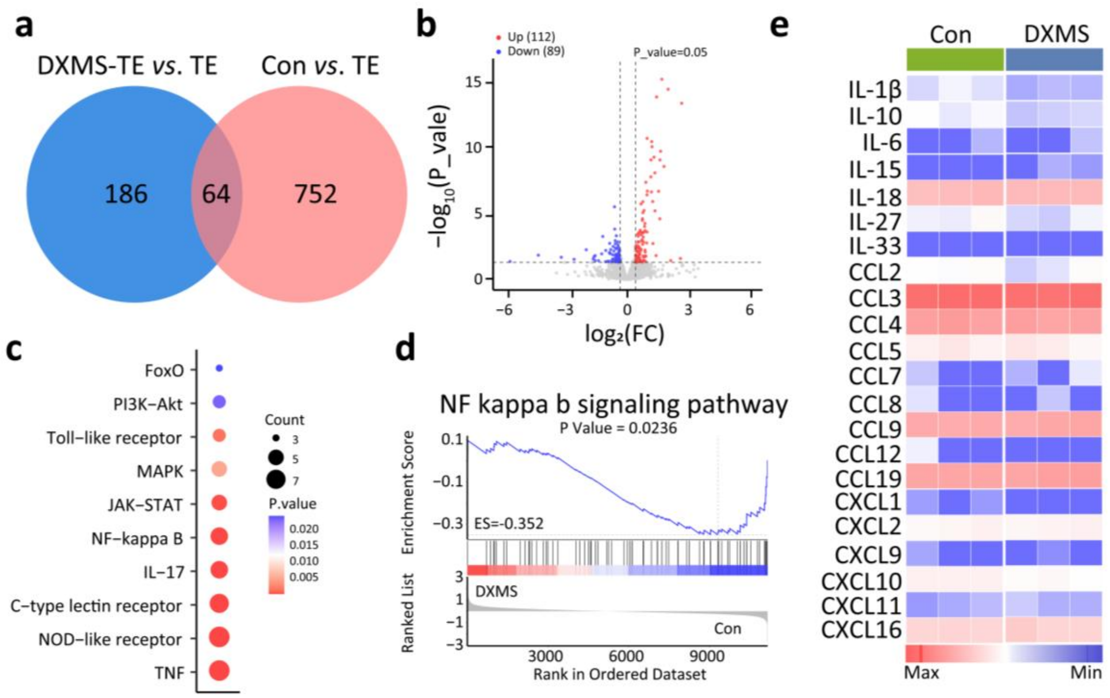

Extended Data Figure 6 | The physiological impact of Dexamethasone on macrophages. a, Venn result of DEGs between DXMS-TE vs. TE and Con vs. TE. **b,** Volcano plot of DEGs in macrophages (n = 3 biologically independent samples). Thresholds: $|\log_2 FC| \geq 0.378$, $p < 0.05$. **c,** Enriched KEGG pathways in macrophages. **d,** Gene set enrichment analysis (GSEA) plot of the NF-κB pathway in macrophages display an enrichment score of -0.352. **e,** Heatmap of 22 differentially expressed cytokine genes in DXMS-treated (DXMS) and untreated (Con) macrophages.

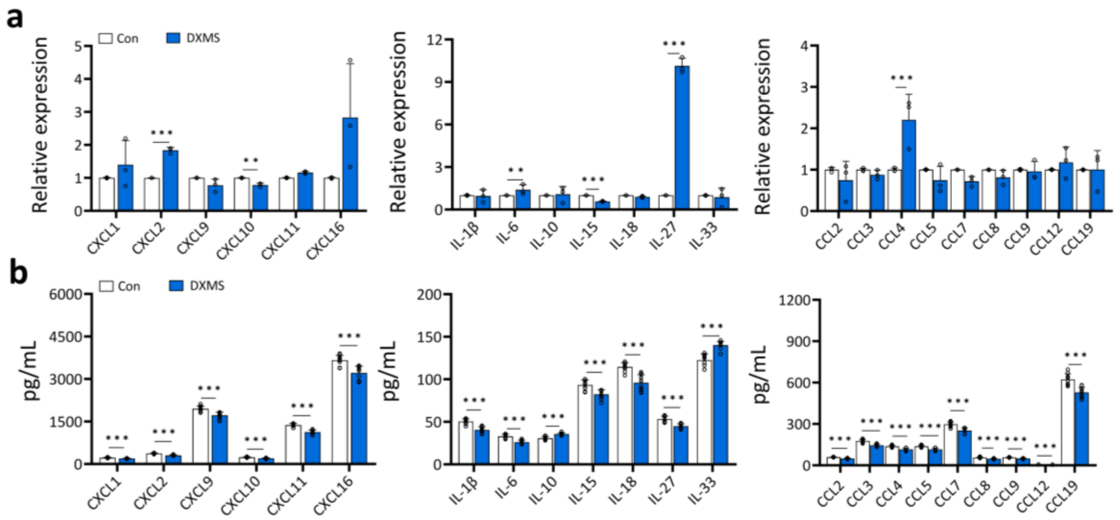

Extended Data Figure 7 | The effect of dexamethasone on cytokine expression in macrophages. a, Quantitative RT-PCR validation on cytokine gene expression after DXMS intervention. Error bars are s.d. (n = 3 biologically independent samples). **b,** ELISA measurements on cytokine gene expression after DXMS intervention. Error bars are s.d. (n = 5 biologically independent samples). *p < 0.05; **p < 0.01; ***p < 0.001 by Student's t test.

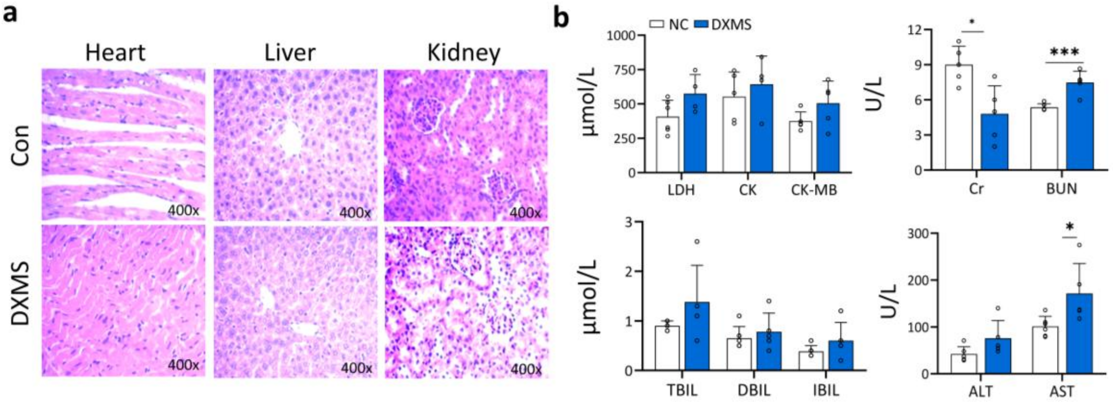

Extended Data Figure 8 | The effect of dexamethasone on physical effect of mice. a, H&E staining of Heart, Liver, and Kidney tissues in DXMS-treated (DXMS) and Control (Con) mice. Magnification × 400. (n = 3 biologically independent samples). **b,** Serum biochemical indicators reveal heart, liver, and kidney functions besides Cr and BUN are not greatly affected after DXMS treatment. Error bars are s.d. (n = 4–6 biologically independent samples). *p < 0.05; **p < 0.01; ***p < 0.001 by Student's t test.

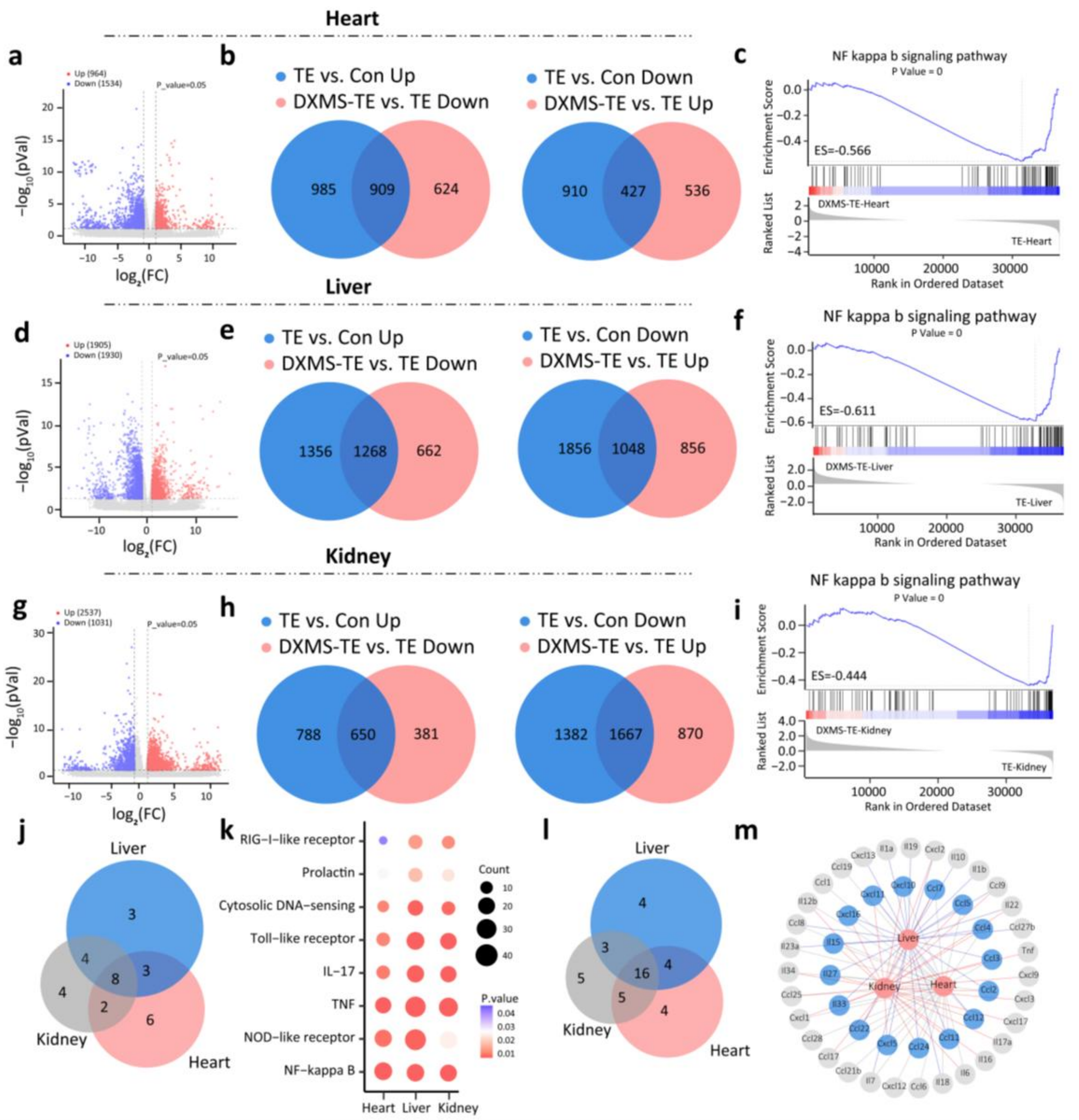

Extended Data Figure 9 | The effect of dexamethasone on the heart, liver and kidney in mice. a, Volcano plot of DEGs in heart. Thresholds: $|\log_2 FC| \geq 0.378$, $p < 0.05$. **b,** Venn result showed 909 DEGs was up-regulated in TE vs. Con and down-regulated genes in DXMS-TE vs. TE, and 427 DEGs was down-regulated in TE vs. Con and up-regulated in DXMS-TE vs. TE. **c,** GSEA plot of the NF-κB pathway in heart display an enrichment score of -0.566. **d,** Volcano plot of DEGs in liver. Thresholds: $|\log_2 FC| \geq 0.378$, $p < 0.05$. **e,** Venn result showed 1268 DEGs was up-regulated in TE vs. Con and down-regulated genes in DXMS-TE vs. TE, and 1048 DEGs was down-regulated in TE vs. Con and up-regulated in DXMS-TE vs. TE. **f,** GSEA plot of the NF-κB pathway in liver display an enrichment score of -0.611. **g,** Volcano plot of DEGs in kidney. Thresholds: $|\log_2 FC| \geq 0.378$, $p < 0.05$. **h,** Venn result showed 650 DEGs was up-regulated in TE vs. Con and down-regulated genes in

DXMS-TE vs. TE, and 1667 DEGs was down-regulated in TE vs. Con and up-regulated in DXMS-TE vs. TE. **I,** GSEA plot of the NF-κB pathway in kidney display an enrichment score of -0.444. **j,** Venn diagram showing the 8 overlapping KEGG pathway in heart, liver, and kidneys. **k,** KEGG pathways co-enriched in the heart, liver, and kidneys. **l,** Venn diagram showing the 16 overlapping cytokines in heart, liver, and kidneys. **m,** The network image shows 8 cytokines across the heart, liver, and kidneys. The inner circle (blue) represents co-upregulated cytokines in Heart, liver and kidneys, the outer circle represents genes enriched in one or two organs. (n = 4 biologically independent samples).

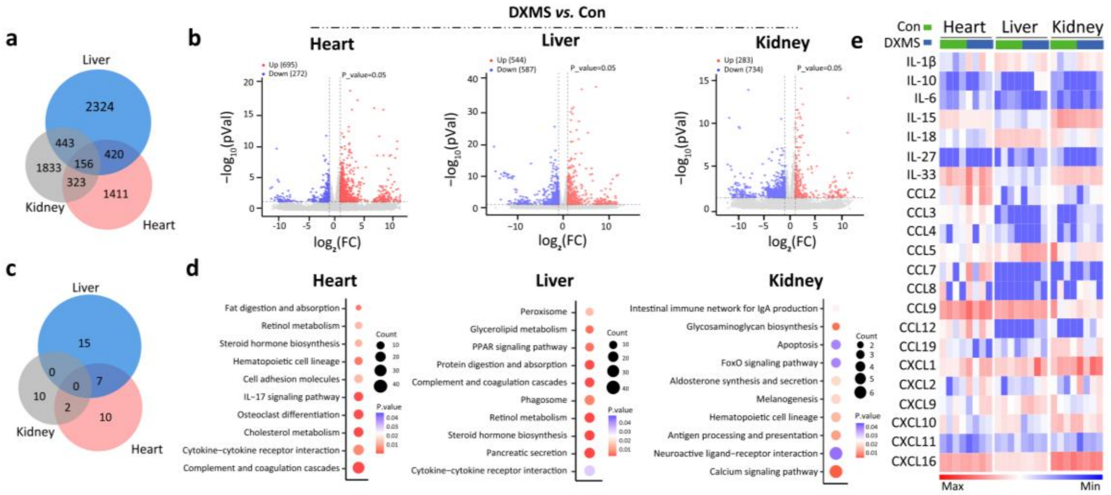

Extended Data Figure 10 | The effect of dexamethasone on cytokine storm in mice. a, Venn diagram of the DEGs in DXMS-treated (DXMS) and Control (Con) mice. **b,** Volcano plot of DEGs in Heart, Liver and Kidney (n = 4 biologically independent samples). Thresholds: $|\log_2 FC| \geq 0.378$, $p < 0.05$. **c,** Venn diagram showing no overlapping KEGG pathway in heart, liver, and kidneys. **d,** The top 10 KEGG pathways enriched by DEGs in Heart, Liver and Kidney, respectively. **e,** Heatmap of 22 cytokine genes in DXMS-treated (DXMS) and untreated (Con) mice. (n = 4 biologically independent samples).

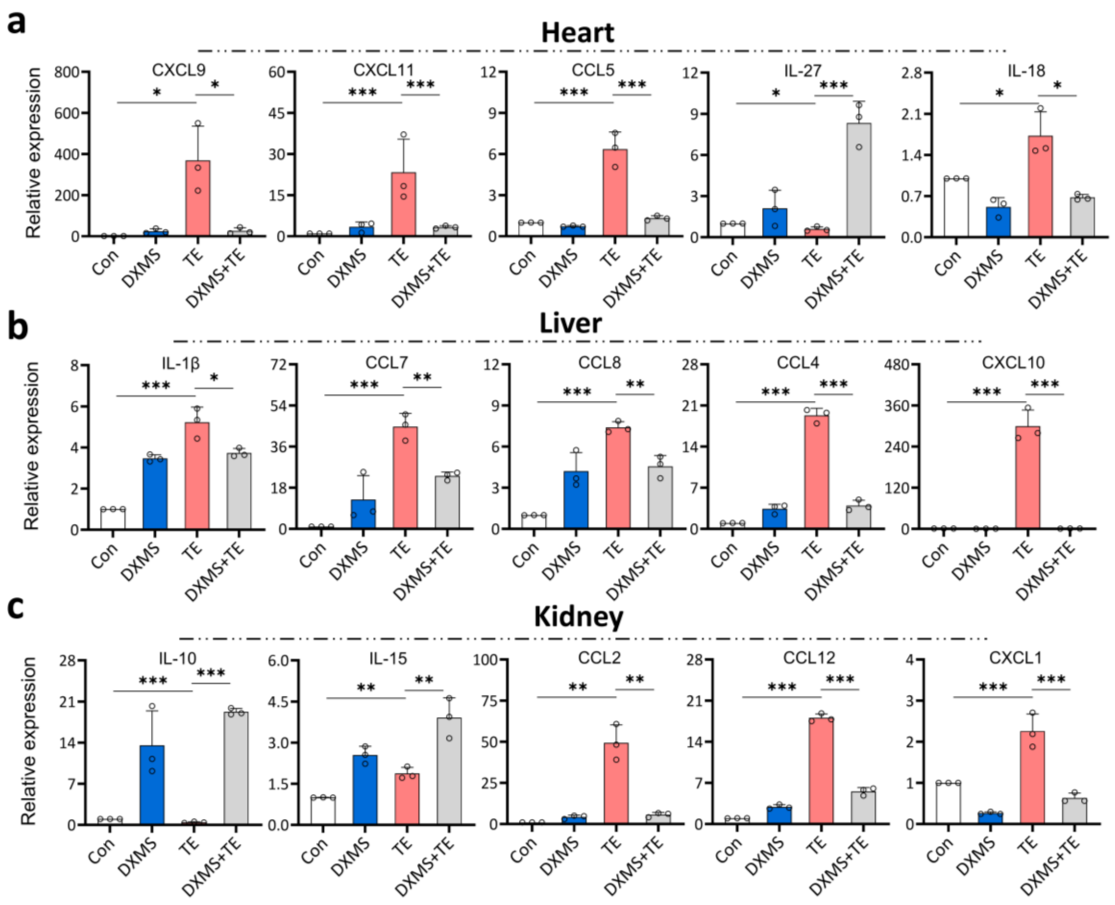

Extended Data Figure 11 | The effect of dexamethasone on cytokine expression on Heart, Liver and Kidneys. a, Quantitative RT-PCR validation dropped five cytokine genes (CXCL9, CXCL11, CCL5, IL-27 and IL-18) expression after DXMS intervention. Error bars are s.d. (n = 3 biologically independent samples). **b,** Quantitative RT-PCR validation dropped five cytokine genes (IL-1β, CCL7, CCL8, CCL4 and CXCL10) expression after DXMS intervention. Error bars are s.d. (n = 3 biologically independent samples). **c,** Quantitative RT-PCR validation dropped five cytokine genes (IL-10, IL-15, CCL2, CCL12, and CXCL1) expression after DXMS intervention. Error bars are s.d. (n = 3 biologically independent samples). *$p < 0.05$; **$p < 0.01$; ***$p < 0.001$ by Student's t test.

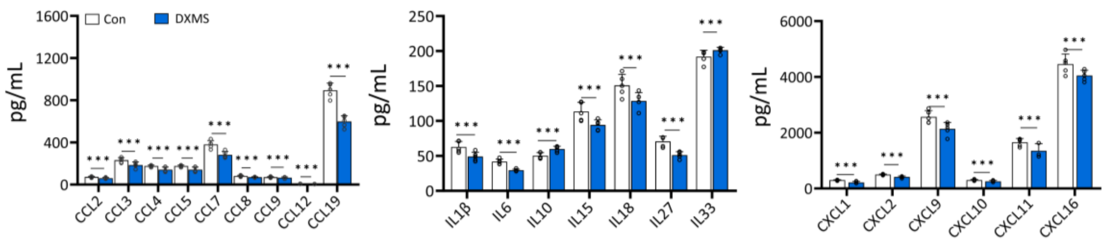

Extended Data Figure 12 | ELISA results showed cytokine protein levels increased in DXMS-treated (DXMS) than Control (Con) group. Error bars are s.d. (n = 3 biologically independent samples). *p < 0.05; **p < 0.01; ***p < 0.001 by Student's t test.
